# Health status assessment of traumatic injury freshwater turtles

**DOI:** 10.1371/journal.pone.0202194

**Published:** 2018-08-28

**Authors:** Alison P. H. Savo, Yaxin Zheng, Yuting Zheng, Gregory A. Lewbart

**Affiliations:** 1 North Carolina State University, College of Veterinary Medicine, Raleigh, North Carolina, United States of America; 2 North Carolina State University, Department of Statistics, Raleigh, North Carolina, United States of America; University of Bari, ITALY

## Abstract

A group of injured yellow-bellied sliders (*Trachemys scripta)* and river cooters (*Pseudemys concinna)* were evaluated for a variety of health values at presentation to the NC State Turtle Rescue Team and prior to release. An i-STAT Portable Clinical Analyzer and CG8+ cartridges were used to determine venous blood gas and biochemical values, the packed cell volume (PCV) and total protein were evaluated using hematocrit tubes and high speed centrifugation, and a differential WBC percentage was determined manually with Diff-Quick stained blood smear slides. Forty-six turtles were sampled on presentation and twenty-three of those were sampled again prior to release. Blood values were analyzed for significant differences between samples collected at presentation and prior to release, as well as differences between surviving and non-surviving turtles. Five variables were identified as significantly different between presenting and recuperated samples: pH, pCO2, Glu, % heterophils, and % eosinophils. When comparing samples between turtles that survived versus those that did not, two variables were identified as being significant prognostic indicators; lactate and PCV. Identification of these significant variables can aid in determining patient prognosis and triage therapy for injured aquatic turtles.

## Introduction

Turtle populations in the United States are in decline due to habitat fragmentation, vehicular mortality, and the expanding pet trade [1, 2, 3]. Human populations continue to grow and encroach on critical habitats for native wildlife. With many turtle species living and reproducing for upwards of fifty years, each individual can have an impact on species conservation. Turtles present to the NC State Turtle Rescue Team (TRT) with viral infections, aural abscesses, and traumatic injuries, which commonly include vehicular trauma, dog attacks, and fishing gear foreign bodies. Treatment plans are developed based on physical examination findings and in some cases radiographic imaging. These methods can be somewhat subjective and a patient’s prognosis can be hard to categorize. This project considered two questions. First, when comparing blood samples collected at presentation versus pre-release recuperated samples, will there be differences in the blood values? Secondly, are there differences in blood values when comparing turtles that survived and were released versus turtles that died in the hospital, and could these values be used as prognostic indicators? We expected to find reliable differences between turtle groups, particularly with lactate and PCV. Comprehensive blood work is not routinely performed for logistical and financial reasons. Reliable blood gas, hematological and biochemical analysis provide a more definitive assessment of health status, and consequently development of a more effective treatment plan. These values might also help determine the prognosis of our patients, which would allow for us to proceed with the most humane option upon patient arrival. A number of papers describe the use of blood gas, biochemical, and hematological analysis to determine the health status of cold-stunned and injured sea turtles [4, 5, 6, 7, 8, 9, 10, 11]; however, little has been done looking at freshwater aquatic and terrestrial turtles. The data collected during this study investigates correlations between certain clinical pathology differences and turtle release rates, ultimately looking for values that could be early prognostic indicators.

## Materials and methods

### Ethics statement

All handling and sampling procedures were consistent with standard vertebrate health care protocols and veterinary practices. All procedures described in this paper involving vertebrate animals were specifically approved by the NCSU institutional Animal Care and Use Committee (NCSU IACUC protocol 16-049-O) in compliance with the Guide for Care and Use of Laboratory Animals 8th Ed.

#### Turtle selection and sampling

A total of forty-six injured turtles were sampled upon intake and twenty-three of these turtles had a second venous blood sample collected from them prior to release. Fourteen variables were examined to compare presentation versus recuperated blood samples. Samples were collected from two species, yellow-bellied sliders (*Trachemys scripta)* and river cooters (*Pseudemys concinna)*. The species were combined in this study to increase sample size. These two species share similar diet and habitat requirements and have been known to hybridize naturally. The turtles were selected (trauma patients not slated for euthanasia) from patients admitted to the TRT. Initial blood samples were collected from injured adult turtles within twenty-four hours of presentation. In some cases patients received fluids and/or analgesics. Sex, clinical presentation, and the time of year were recorded and taken into consideration when analyzing the biochemical data. These factors have been shown to impact normal blood values [12, 13, 14].

Cloacal temperature data were collected using a thermocouple thermometer (model EW-91219-40; Cole-Parmer, Vernon Hills, IL, USA) just prior to blood collection (Table 1). Body weight was measured in grams using a digital scale. The sex was determined by external sexual dimorphism; primarily nail length on the forelimbs and vent distance along the tail. The species were distinguished by identifying the yellow-bellied slider’s characteristic black spots on the gular scutes or by comparison of carapace shape. River cooters have a large, more oval, carapace compared with sliders.

**Table 1:** Descriptive statistics of NC State turtle rescue research.

Variable	Blood test 1 (n = 46)		Blood test 2 (n = 23)	
	Mean	SE	Mean	SE
Sex	23 Males^1^ 22 Females	0.51	Didn’t measure second time	
Weight	1608.73	915.97	Didn’t measure second time	
Temperature	22.01	2.02	Didn’t measure second time	
PCV	16.13	8.12	17.83	5.40
TP	4.41	4.13	3.92	1.26
Lactate	5.06	5.98	1.37	1.08
pH_A_	7.55	0.20	7.90	0.14
pH_M_	7.04	1.85	6.83^2^	2.65
pCO_2A_	33.65	23.12	19.74	5.00
pCO_2M_	31.03	13.19	20.39	5.36
pO_2A_	41.85	36.09	76.40	29.83
pO_2M_	73.50	40.66	115.43	31.58
Na	128.13	9.67	127.6	7.44
K	3.82	1.21	2.90	0.53
iCa_A_	0.96	0.56	0.98	0.46
iCa_M_	1.12	1.16	0.89	0.41
Glu	148.83	105.72	55.30	24.55
WBC Count	9104.55	6166.45	8640.00	3420.43
% Heterophils	62.18	18.83	43.75	23.01
% Eosinophils	15.70	14.57	25.25	23.49
% Lymphs	12.82	8.02	16.85	9.38
% Basophils	0.50	2.72	0.50	1.19
% Monocytes	3.52	4.66	8.20	6.65
% Azurophils	5.23	4.83	5.35	4.98

Note

1. Only 45 turtles have sex values. One turtle missing sex value.

2. Calculation based on first measurement temperature since turtle’s body temperature to be the fairly static.

### Blood sample collection

Blood samples (approximately 0.3 mL) were collected through venipuncture from the right brachial vein with a heparinized 1-ml syringe and 25-gauge needle. If a blood sample was not collected from the brachial vein due to injury, the dorsal coccygeal vein was used. All blood samples were analyzed within ten minutes of collection. Any turtle that recovered to the point of release had all of the clinical tests repeated. The second blood samples were collected no earlier than three days prior to release.

#### Venous blood gas and biochemistry values

An i-STAT Portable Clinical Analyzer (Heska Corporation, Fort Collins, Colorado, USA) with CG8+ cartridges were used to test pH, partial pressure oxygen (pO2,), partial pressure carbon dioxide (pCO2), bicarbonate (HCO3-), hematocrit (Hct), hemoglobin (Hb), sodium (Na), potassium (K), ionized calcium (iCa), and glucose. The iSTAT analyzed the blood at 37°C, then corrected pH, pCO2, and pO2 based on individual body temperature once that information was entered. We also manually calculated corrections for mean pH, pO2, pCO2, and iCa, based on the turtle cloacal temperature (Ti) at the time of sampling using the equations listed below. Both sets of values (i.e., those derived from auto-corrections from the iSTAT and those derived from independent calculations) are reported in Table 1. Lactate was measured using a Nova Lactate Plus (Nova-Biomedical, Waltham, MA, USA) hand-held analyzer.

$$\begin{array}{l} pH_{M}=0.104(\Delta T_{37-25})+0.005(\Delta T_{25-Ti})+pH_{1}[at\;temperatures<25{}^{\circ}C]\\ pO_{2M}=pO_{12}(10_{-0.0058\Delta T})\\ pCO_{2M}=pCO_{21}(10_{-0.019\Delta T})\\ iC_{aM}=iC_{aI}(1+0.53[pH_{1}-pH_{M}] \end{array}$$

### Hematology

Packed cell volume was measured using blood filled hematocrit tubes and high-speed centrifugation. Total solids were measured from plasma with a standard refractometer. Blood smears were prepared with a Diff-Quick (Jorgenson Laboratories, Loveland, CO, 80538 USA) stain and read using a light microscope at a later date. Differential white blood cell percentage was performed using 100 white blood cells on the fixed blood smears. The estimated white blood cell counts were obtained by counting 10 40X objective fields, averaging the number, and multiplying by 2,000 [15].

### Statistical analysis

Due to the variability of reptilian blood values and limitation of instrument sensitivity, some values were not detectable. The minimum threshold is used for those data points that were recorded as beyond measurable. For instance, if the value of iCa was recorded as <0.25, 0.25 was used in the statistical analysis. Zero is used to substitute missing values.

To compare the differences in blood value measurements between the initial sample and the prerelease sample a Hotelling’s T2 test was performed. That analysis was performed in SAS 9.4 (SAS Institute, Cary, NC) and RStudio version (1.0.136). Assumptions of independence among the turtle samples, equal covariance, and normal distributions were made. Data were visually inspected, t-test are robust against violations of normality. No severe skewness and outliers were observed.

A multiple logistic regression analysis was performed to investigate the likelihood of survival based on certain blood values. The control for multicollinearity, weight, K, and % heterophils were not analyzed due to their high correlation with other variables. Multicollinearity exists if two or more of the predictors in a regression analysis are moderately or highly correlated, which means some predictors are linear combinations of others. Multicollinearity biases our estimates and limits the conclusions. A Hosmer-Lemeshow (HL) test was used to evaluate goodness of fit for this logistic regression model. Bonferroni simultaneous confidence intervals were generated for the five significant variables. An ROC curve of the multiple logistic regression model prediction was calculated; this curve measures the accuracy of the model.

A decision tree model divided the dataset into a training dataset (n1 = 35) and a validation dataset (n2 = 11). The target variable for the training dataset was survivability, y = 1 for survival, and y = 0 for death. The validation dataset was created by applying prediction rules to calculate the percentage of the correct prediction of survival.

## Results

The four variables with significant differences between the two measurements were pH, pCO2, glucose, and % heterophils. The results are summarized in Table 1. Of the original forty-six turtles, seventeen died and six were released prior to the second sample collection. Two of those six turtles were hospitalized for only twenty-four hours, and the remaining four were lost to follow-up while in off-site rehabilitation. There were fifteen male YBS, twelve female YBS, one juvenile YBS, one red bellied cooter, seven male cooters, and ten female cooters sampled. The length of hospitalization and rehabilitation time varied for each turtle based on the severity of the presenting injury. The average length of hospital stay for released turtles was thirty-two days, with the maximum stay being one hundred days, and minimum being one day. Of the seventeen turtles that died, the average hospitalization time was fourteen days, with the maximum stay being fifty days, and minimum stay being less than twenty-four hours.

The most common presenting injury was vehicular trauma, accounting for forty-one of the forty-six turtles. The remaining five turtles presented with oral fish hook foreign bodies. All seventeen of the turtles that died had hit-by-car injuries. 58.8% had an obvious coelomic breech, 41% had spinal trauma, and 17.6% had palpable facial fractures. The injuries of the surviving turtles were comprised of predominantly minor carapace fractures; only 8% (2 turtles) had an obvious coelomic breech.

### Hotelling’s T2 test

**Fig 1:**
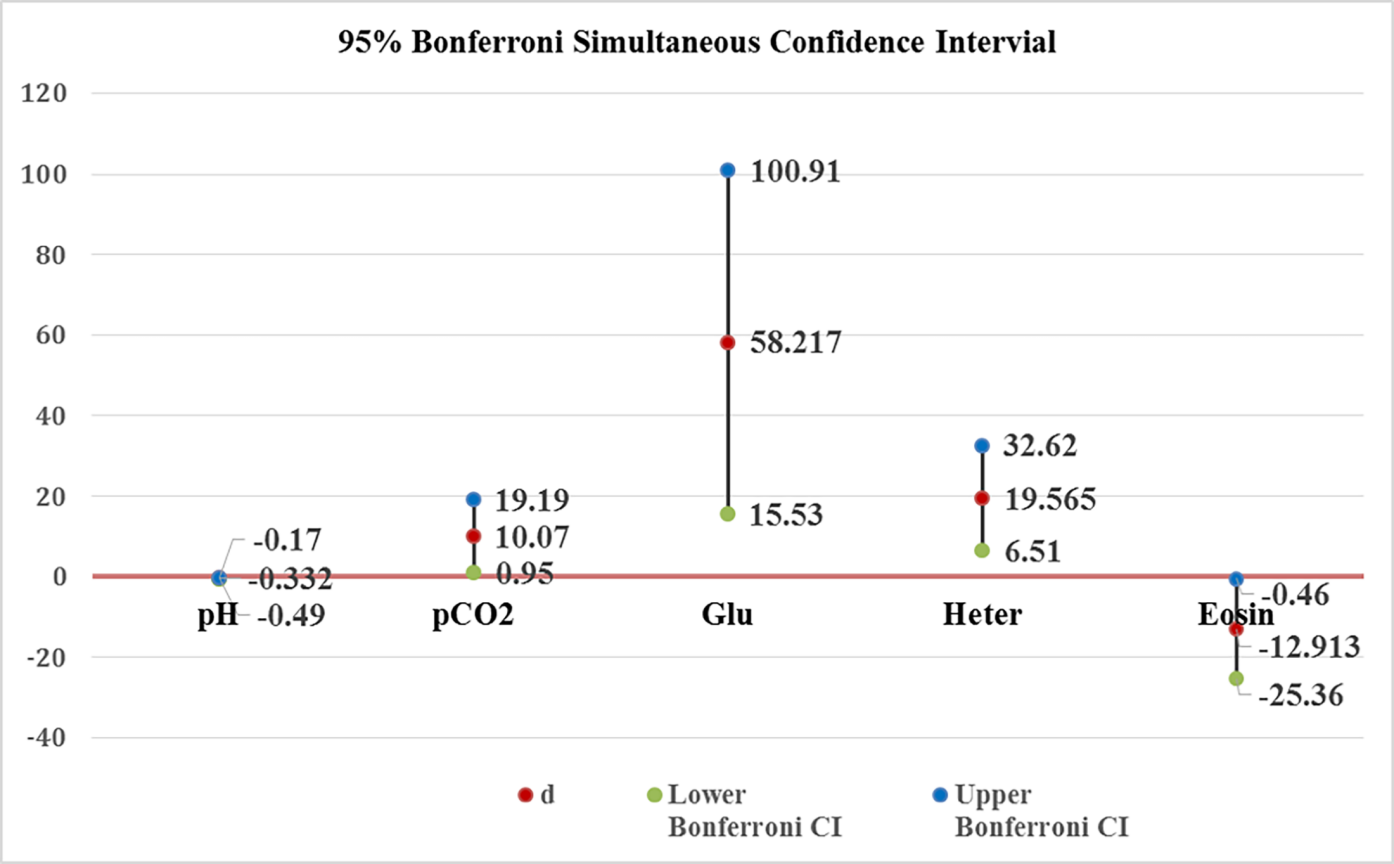
95% Bonferroni simultaneous confidence interval for significant variables.

Table 2 gives the results from the Hotelling’s T2 test. Four variables (pH, pCO2, Glu, % heterophils) have significant differences between the two measurements. The Bonferroni simultaneous confidence intervals for the five significant variables are shown in Table 3 and a visual presentation in Fig 1.

**Table 2:** Hotelling’s *T*^*2*^ test summary.

Hotelling’s *T*^*2*^ test	F	df1 (p)	df2 (n-p)	p-value
869.045	17.242	14	5	0.0026798

Note: d represents the averages of the difference of blood test 1 –test 2 for each variable.

**Table 3:** Results of 95% Bonferroni simultaneous confidence intervals.

Variable	*d*	Lower Bonferroni CI	Upper Bonferroni CI
PH	-0.264	-0.42	-0.1
PCO2	13.336	1.95	24.72
Glu	51.15	8.76	93.54
% Heterophils	18.3	4.36	32.24

Note: d represents the averages of the difference of blood test 1 –test 2 for each variable.

### Logistic regression

The stepwise selection procedure resulted in five variables for the logistic regression model: sex, lactate, PCV, temperature and pO2. The multiple logistic regression results are presented in Table 4.

**Table 4:** Logistic regression output.

Variable	*b*	SE	*P*-value	Odds Ratio	Inverse Odds Ratio
(Intercept)	3.214	7.069	0.649	24.879	
Sex	-0.959	0.704	0.173	0.383	2.611
Temperature	-0.348	0.328	0.289	0.706	1.416
PCV	0.228	0.103	0.027**	1.255	
Lactate	-0.300	0.151	0.047**	0.741	1.350
PO2	0.051	0.028	0.067*	1.053	

Note

*p < .10.

**p < .05.

***p < .01.

**Fig 2:**
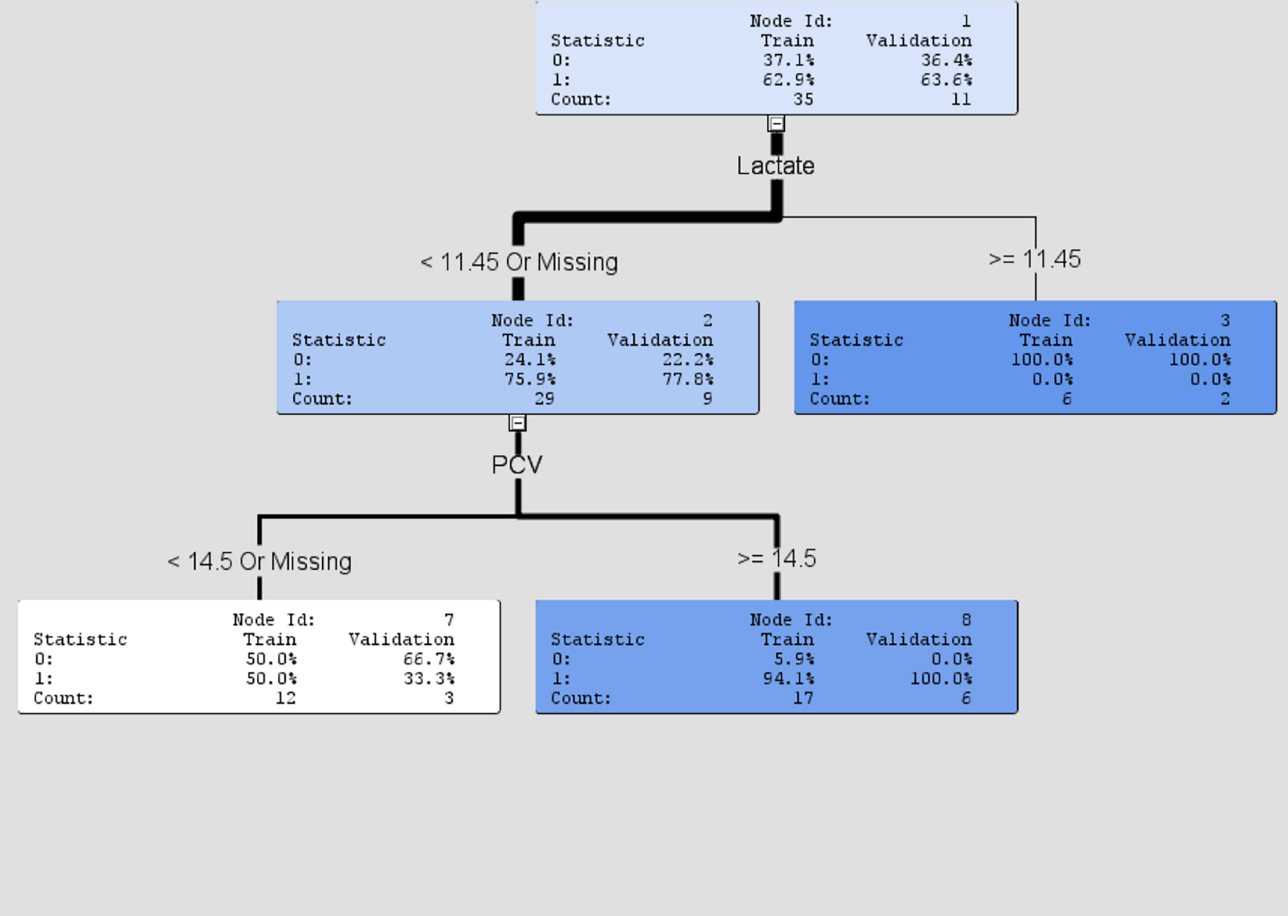
ROC Curve for multiple logistic regression model.

Packed cell volume had a strong positive and significant association with turtle survival (*b* = .228; *p*-value = .027). As PCV increased by one unit, the estimated odds of survival increased by a factor of 1.255, while holding other variables constant. A unit increase in lactate lowered the odds of survival by 1.35. Moreover, a unit increase in PO2 increased the odds of survival by 1.053. The area under the ROC curve is 0.9418, indicating that this regression model has high accuracy in predicting turtle’s survival and death (Fig 2).

### Decision tree

**Fig 3:**
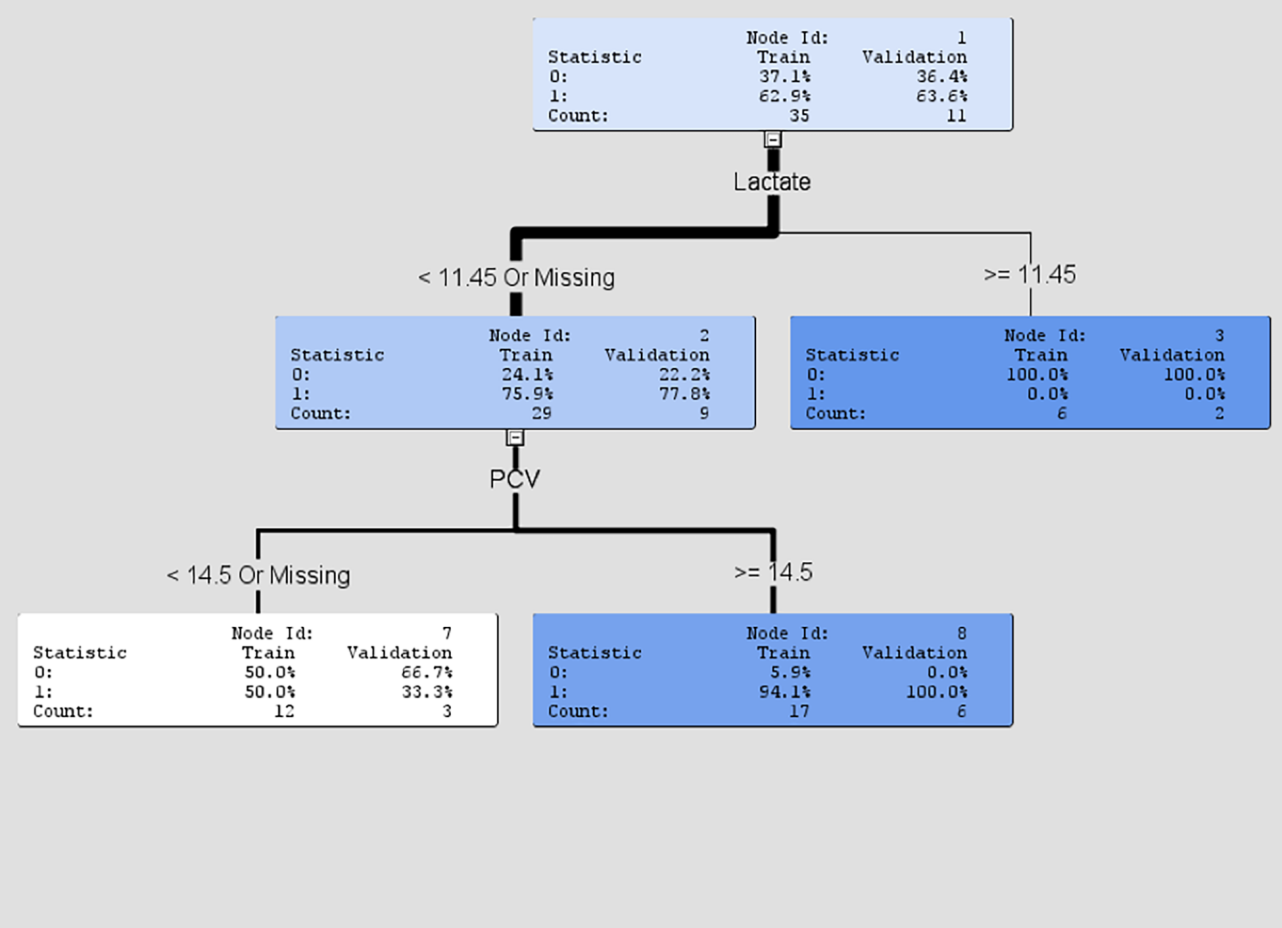
Decision tree output.

The decision tree was based on lactate and PCV variables. These variables were determined to be the most important predictors of survivability through the logistical regression model. Of the turtles with a lactate lower than 11.45mg/dL (or a missing sample), 75.9% survived in the training dataset and 77.8% in the validation dataset. Among them, using PCV as the second splitting node, 94.1% proportion of the turtles with PCV greater than or equal to 14.5% survived, and 100% in the validation dataset. The results of the decision tree model are given in Fig 3.

## Discussion

This study evaluated of the effectiveness of blood gas, biochemical and hematological analysis in determining the physiological status of sick and injured turtles. The iSTAT values affected by temperature (pH, pCO_2,_ pO_2_, and iCa) were both auto-corrected by the iSTAT and manually corrected with a published, standard formula. This was done because other investigators have noted slight differences when this level of accuracy is employed [16, 17, 18]. The differences between the automated and manual corrections were not clinically significant. The remainder of the discussion references the automated corrections because they will be most accessible in a clinical triage situation.

Biochemical changes associated with shock include hyperlactemia and acidemia, therefore, a decreased pH at presentation was expected due to the severe injury and presumed hypovolemia seen in our patients [19]. The pCO2 decreased by an average of 10.72mmHg to reach an average of 19.91mmHg among all of the released turtles. This change in CO2 concentration is further evidence of the acidemia and respiratory acidosis of the injured turtles at presentation. We did not see a significant change in O2 when comparing presentation samples to recuperated samples. Reptiles have an incomplete interventricular septum; intracardiac left-right-shunting increases the oxygen transport systemically [19]. This could explain why the blood O2 levels were not as affected as other values.

Like other species, turtles have an increased blood glucose during times of stress. Several studies investigating the blood biochemistry of sea turtles found that glucose increased significantly after capture or transport [6, 13, 20, 21]. Hyperglycemia was reported in one red-eared slider (*Trachemys scripta elegans*) with nematode-induced pancreatitis [22]. The average glucose at admission for all forty-six turtles in this study was 148mg/dL. The normal value of glucose for red bellied sliders is 74mg/dL [13], and for free ranging western pond turtles is 68mg/dL [9]. Based on these established normal medical reference intervals the blood glucose concentration for our turtles at presentation was significantly elevated. For the twenty-three turtles that were released, the glucose dropped by an average of 58mg/dL, reaching a mean of 54mg/dL, which falls within the expected normal reference value. These findings indicate that glucose could be used as a quantitative measurement of stress among fresh-water turtles.

Heterophils are the predominant cell type induced during inflammation and they work to control microbial invasion [23]. The average heterophil count from all forty-six turtles on presentation was 62%. The heterophil count decreased by 20% in the recuperated sample among the turtles that were released. Normal heterophil counts for fresh-water turtles vary considerably, 9% in red-bellied cooters [13], 53% in Mediterranean pond turtles [22], 20% in western pond turtles [9], and 44% in box turtles [24]. We suspect that 62% heterophils is an elevated value. The turtles investigated in this study all sustained some degree of trauma, so an inflammatory response is expected. Based on these comparisons the inflammatory response declined among the recuperated turtles. In mammals eosinophils play a role in hypersensitive reactions and parasitic infections [23]. Within a group of free ranging western pond turtles the average eosinophil count was 31.6%, and among a captive population of the same species the eosinophil count was 16.5% [25]. This could indicate that eosinophils play a role in parasitic control in reptiles as well. The average eosinophil count was 15.5% for all forty-six turtles. Of the turtles that were sampled prior to release, the eosinophil count increased an average of 12.9%, to reach an average of 25.3%. Much like heterophils, the reference values for eosinophils vary a great deal in the literature [9, 13]. Considering the eosinophil count increased with increasing health status, it can be hypothesized that a higher eosinophil count is normal for these species. The remaining white blood cells (lymphocytes, basophils, monocytes and azurophils) were not significantly different between presenting and pre-release samples. While this data did not prove useful as prognostic indicators, it may aid to further establish normal reference ranges in these species.

According to the logistical regression model, lactate and PCV are the strongest prognostic indicators for the turtles in this study. Of the turtles with an initial lactate lower than 11.4, 75.9% survived to release. Of the turtles with a lactate greater than 12 100% died (seven turtles were in this category). The most common cause of hyperlactemia in veterinary patients is shock [26]. Many of the turtles in this study sustained life-threatening physical trauma. In some cases the severity of the injuries may have caused hypovolemia, low cardiac output, and septicemia, resulting in inadequate oxygen delivery and increased anaerobic metabolism. Oxygen is required for lactate to be metabolized and cleared from the bloodstream. Lactate is not a novel prognostic indicator. In cold-stunned Kemp’s ridley turtles increasing lactate levels were correlated with non-survival [25]. In dogs, lactate has some prognostic value in critically ill and injured individuals, specifically when dealing with gastric dilation and volvulus, babesiosis, severe soft tissue infections, abdominal evisceration, and septic peritonitis [26]. In mammals a lactate value above 2 is considered a poor prognostic indicator in most cases. Reptiles have a much higher tolerance. It is known that lactate plays a similar role in sea turtles; an entanglement time of 30 minutes or more results in an elevated plasma lactate [21]. Though physiologically sea turtles and fresh-water turtles have important differences, the results of this study suggest that lactate may be of prognostic value in fresh water turtles. Interpretation of lactate values outside of injury or diseases states should be approached with caution. Lactate levels can be extremely high in anoxic turtles during hibernation [27], with a value of over 200 mmol/L recorded in the painted turtle, *Chrysemys picta* [28]. Members of the Chelydridae and Emydidae appear to be the most tolerant on lactate levels, with some species able to tolerate up to eight times the values in mammals [28]. In mammals excess lactate (following exercise) is reduced by oxidation while in fishes and lizards glycogen synthesis is responsible [28]. The exact mechanism for lactate removal in turtles is unknown, but, may have oxidation as a component [28].

Packed cell volume is the second prognostic indicator identified in this study. In the decision tree model (Fig 3), turtles that had a lactate lower than 11.45mg/dL were further analyzed for survivability using PCV. Of the turtles within this group that presented with a PCV greater than 14.5%, 94.1% survived to release. Those turtles that experienced severe hemorrhage, resulting in a low PCV, had a decreased survival rate. The established normal hematocrit value for some freshwater turtles is approximately 19–25% [9, 13, 22, 29]. The average PCV among the twenty-three recuperating samples was 18%, very similar to other established reference values. On average turtles returned to a normal PCV prior to release.

Triage is an important component of wildlife medicine and having a method to determine which patients have the best chances of survival is a priority. Performing a PCV and measuring lactate uses a very small volume of blood, can be done in the clinic or field, and are relativity inexpensive compared to a full CBC and blood biochemistry analysis. When trying to determine if treatment should be pursued, or if euthanasia is the most humane option, these tests provide some insight into the health status of the patient allowing for a more informed decision.

## Supporting information

S1 Data Harrelson et al. (XLSX)
